# Metabolic syndrome – a risk factor for all-cause disability pension: a prospective study based on the Swedish WOLF cohort

**DOI:** 10.5271/sjweh.3881

**Published:** 2020-07-01

**Authors:** Edvard Lidén, Berndt Karlsson, Kjell Torén, Eva Andersson

**Affiliations:** Department of Occupational and Environmental Medicine, Sahlgrenska Academy, University of Gothenburg, Gothenburg, Sweden; Department of Public Health and Clinical Medicine, Umeå University, Umeå, Sweden; Department of Occupational and Environmental Medicine, Sahlgrenska University Hospital, Gothenburg, Sweden

**Keywords:** Key terms abdominal obesity, blood lipid, employee, glucose, hypertension, longitudinal, Sweden, work ability

## Abstract

**Objective:**

The aim was to study the impact of metabolic syndrome on the risk for disability pension among Swedish employees.

**Methods:**

A working population-based prospective cohort [Work, Lipids and Fibrinogen (WOLF) cohort, N=10 803], was linked to national registry records of all-cause disability pension for the period 1992–2013. Occupational health service data included 1992–2009 anthropometric measurements, blood samples, and questionnaires. Metabolic syndrome was defined according to International Diabetes Federation criteria, and risk for any all-cause disability pension was analyzed using Cox proportional hazard regression as hazard ratios (HR) with 95% confidence intervals (CI) adjusted for age, sex and other covariates.

**Results:**

Of the employees, 17.9% (men 21.5%, women 9.7%) met the criteria for metabolic syndrome. The prevalence of all-cause disability pension was 15.2% in men with metabolic syndrome and 7.5% in men without metabolic syndrome; for women, the corresponding results were 23.2% and 12.7%. After adjustment for socio-demographic factors, health behaviors, work-related factors, diabetes, and obesity, the risk for all-cause disability pension among subjects with metabolic syndrome displayed an HR of 1.37 (95% CI 1.18–1.60). Results were similar for men and women. In a subgroup, further adjustment for chronic diseases resulted in an HR of 1.32 (95% CI 1.04–1.68).

**Conclusion:**

This study demonstrates an increased risk for all-cause disability pension, even after adjustment for other risk factors, among Swedish employees with metabolic syndrome compared to those without at baseline.

In 1998, the World Health Organization (WHO) defined simultaneous occurrence of several cardiovascular risk factors – insulin resistance, dyslipidemia, hypertension, obesity or central obesity (increased waist circumference) – as metabolic syndrome (MetS) (1, 2). Several definitions of MetS are valid, each focusing on different metabolic factors in the syndrome. The prevalence of MetS in different European and North American populations is estimated to be 9.4–23.8% depending on the criteria definition of MetS and the observed population (3, 4). Diabetes mellitus type 2 (DM2) and MetS are separate disorders but could both be associated with cardiovascular comorbidity as they share similar metabolic risk factors; there are also indications that MetS in itself could be a precursor of cardiovascular disease and DM2 (5–7). Published data describing work ability and disability pension in diabetic patients in the workforce are numerous (8–11) but there is considerably less, if any, knowledge about the impact of MetS on work ability and the risk for disability pension. Of relevance is a study by Lam & LeRoith (12) describing the multi-organ effects related to MetS, with insulin resistance playing a key role in the development of MetS and another by Taylor & MacQueen (13), who in 2007 described the effect on cognitive performance in MetS.

The concept of work disability encompasses several areas of science but its endpoint – disability pension – is a statutory or insurance-based construct that depends on identifiable medical diagnoses relating to biological characteristics of an individual. Medically motivated impairments lead to an incapacity of the individual to perform allotted work tasks and maintain gainful full-time employment until retirement age (14). Work ability can be evaluated with prognostic tools such as the Work Ability Index (WAI), but there is a lack of methods for assessing work disability in epidemiological studies (15). The possibility to predict the risk for disability pension has been described in a follow-up study of work-related psychosocial demands (16). Sustained work disability may eventually lead to disability pension, which can be monitored and evaluated using social registry data on disability pension (17).

The Work, Lipids and Fibrinogen (WOLF) cohort is well studied (18). The WOLF surveys are based on questionnaires, clinical examinations, anthropometric measurements, and blood sampling among employees in different branches of work and different parts of Sweden during 1992–2009 and, therefore, provides valuable data for the present study (www.wolfstudy.se).

The aim of this study was to explore the impact of MetS on the probability of disability pension among employees in the Swedish workforce.

## Methods

### Design and study population

We conducted a working population-based prospective cohort study using data collected since 1992 from the WOLF cohort surveys. Study subjects participated in an extensive set of WOLF surveys comprising anthropometric measurements and blood samples at baseline and questionnaires administered on one to three occasions during 1992–1997 and 2000–2003, with a follow-up survey in 2009. The study subjects were 15–64 years of age and were recruited from 36 occupational health service units and employed in 159 different occupations (according to the Nordic Classification of Occupations (19) in approximately 60 companies located in Stockholm county and in the northern Swedish counties of Västernorrland and Jämtland. Workers in the enrolled companies were invited to participate, but subjects on long-term leave from their workplace were not included. Participating workers were employed in transportation, industry, public administration, telecommunications, sales, teaching, construction and finance.

Clinical examination at the occupational health service units included standardized measurement of blood pressure (BP) and measurements of length, weight, and waist and hip circumference. Sampling of fasting blood glucose (FPG) and blood lipids followed standardized procedures and the blood samples were analyzed at an accredited laboratory. The WOLF survey questionnaires addressed work and health conditions, socio-demographic status, and job strain and other psychosocial factors. The questionnaires also included questions on tobacco and alcohol use, exercise habits, and cardiovascular risk factors.

For 10 803 study subjects derived from the WOLF cohort, complete information on parameters regarding MetS was available. In the northern Swedish counties, 2013 subjects completed a second clinical investigation. All participants in earlier WOLF surveys were invited to respond to the 2009 follow-up survey, 6352 subjects answered and thus formed a sub-group. The final 2009 survey also included questions regarding specific chronic diseases.

### Definitions

*Disability pension*. We chose to use any all-cause disability pension (full- or part-time) as a proxy for work disability. The WOLF cohort data were matched with de-identified data from an official registry, the Longitudinal Integrations Database for Health Insurance and Labor Market Studies (LISA) registry, published by Statistics Sweden (SCB). The LISA register is updated yearly and comprises aggregated socio-economic and demographic data, derived from different registers, on all citizens aged >16 years living in Sweden (www.scb.se). The LISA database is enabled by the use of unique personal identification numbers (PIN) (20). In this study, the matching LISA data cover the years 1990–2013, and the subjects were followed regarding all-cause disability pension from their first year in the study until the last year of the follow-up. Statistics Sweden carried out the merging of data for all subjects in the WOLF cohort with corresponding data from the LISA database. The merged data for the study period 1990–2013 were then anonymized (ie, the PIN were removed) and sent back for further analysis.

*Metabolic syndrome*. Of the different definitions of MetS, we preferred using the definition given in 2005 by the International Diabetes Federation (IDF) (www.idf.org) (21), for analysis of the WOLF survey data. The IDF criteria for the MetS require the mandatory presence of central obesity (a circumferential waist measurement of >94 cm for men and >80 cm for women) and the presence of >2 of 4 of the following criteria: (i) raised FPG ≥100 mg/dL (5.6 mmol/L) or diagnosed diabetes; (ii) raised BP, with systolic BP >130 or diastolic BP >85 mmHg, or treatment for diagnosed hypertension; (iii) raised triglycerides ≥150 mg/dL (1.7 mmol/L) or pharmaceutical treatment for blood lipids; and (iv) reduced level of high-density lipoprotein cholesterol (HDL-C), of <40 mg/dL (1.0 mmol/L) in males and <50 mg/dL (1.3 mmol/L) in females, or pharmaceutical treatment for blood lipids. Information on use of pharmaceutical treatment and diabetes diagnosis was derived from the questionnaires.

The study population was divided into two groups, those with MetS at baseline or at the second clinical investigation (MetS group) and those without metabolic syndrome (non-MetS group). If a study subject met the MetS criteria at the second medical investigation, this occasion constituted baseline in the further analyses.

*Socio-demographic factors*. Region was classified according to employment location as 0=Stockholm county, and 1=the counties of Västernorrland and Jämtland. Job level was derived from the LISA registry, and 10–13 occupationally based socio-economic groups (the division of groups was changed by the SCB during the follow-up time) were categorized into three levels. In the final analyses, levels 2 and 3 were combined (as their results were similar) and compared with the highest job level (level 1). Classification of education was done based on the baseline questionnaire, where one item covered nine types of school as well as university education. Educational level was divided into low (6–9 years) and high (university or similar), both of which were compared with medium-length education. Civil status was derived from the baseline questionnaire, with four response alternatives (married or cohabiting/unmarried/divorced/widowed) classified as 0=married or cohabiting, while 1=single.

*Health behaviors*. Smoking habits were derived from items in the baseline questionnaire and categorized as ever-smoking or not. Physical exercise was assessed from the baseline questionnaire item “How often do you exercise?” The item had four response alternatives (never/rarely/sometimes/regularly), with “never” and “rarely” classified as low exercise level and “regularly” as high exercise level. “Sometimes” was used as the reference value.

*Work-related factors*. Level of control was classified according to the demand–control model (22) with five items on demand and six on decision latitude (control) derived from the baseline questionnaire. The items had four response alternatives (yes, often/yes, sometimes/no, rarely/no, never) scoring 1–4 points. The sum was calculated, and the median was used to classify high demand and low control, respectively, as 1. High strain (defined as having both high demand and low control) was also tested but it was low control that demonstrated the strongest association with disability pension and that was therefore used in the analyses. Physical workload at work was classified from the baseline questionnaire, the subjects were asked to assess how physically demanding their work was on a scale of 0=very, very light to 14=very, very strenuous. We classified 0–3 as low and 9–14 as high physical workload and compared them with moderate physical workload of 4–8.

*Metabolic syndrome-related factors*. Diabetes was derived from the baseline questionnaire question “Do you have diabetes?” (yes/no). Body mass index (BMI) was calculated from baseline measurements of weight and length. A BMI >30 was defined as obesity (23).

*Chronic diseases*. From the questionnaire administered at follow-up in 2009, the item “Do you have or have you had any physician-diagnosed diseases or disorders during the last 5 years?” – with specific disease alternatives and three answer alternatives for each disease/disorder (no, not at all/not now, but during the last 5 years /yes, now) – was used to classify “no, not at all” as 0 and the other two responses as 1. These diseases were included in the analysis as they could affect work ability: rheumatologic diseases, musculoskeletal disorders, cardiovascular diseases, psychiatric disease, and asthma/obstructive lung disease.

### Statistical analysis

Frequencies of baseline characteristics, such as MetS criteria and potential risk factors (covariates) for all-cause disability pension used in the analyses, were calculated for all subjects, and for men and women in the MetS and non-MetS group, respectively. For continuous variables, mean values with standard deviations (SD) were calculated. Time at risk (in person-years) was calculated from entry into the study between 1992–2003 (94% of the subjects were included at the end of 1997) until the end of follow-up. End of follow up was the first occurring event of: (i) disability pension, (ii) death, (iii) unavailable in the LISA registry (not registered as a resident in Sweden at the last day of the year, 2.5%), (iv) retirement at age 65 or before, or (v) the end of the study period in 2013. Incidence rates per 1000 person-years, with 95% confidence intervals (CI), were calculated for first occurrence of all-cause disability pension during time at risk for men and women in the MetS and non-MetS groups, respectively. The risk for all-cause disability pension due to MetS was analyzed using Cox proportional hazard regression in a base model (model 1) adjusting for both age (in years) at study entry and sex, yielding hazard ratios (HR) with 95% CI. Further, three models were analyzed adjusting also for three different sets of covariates: model 2 – base factors and socio-demographic factors (region, job level, education level – low and high compared with medium-length education – and civil status), model 3 – base factors and health behaviors (ever-smoking, physical exercise – low and high exercise level, compared with exercising “sometimes”) and model 4 – base factors and work-related factors (low control in the demand-control model, physical workload at work – low and high workload compared with moderate physical workload). In model 5 (non-MetS-related factors), all three sets of covariates were analyzed together with the base model. In model 6, the base model was analyzed together with the MetS-related factors diabetes and obesity. The final model, model 7, adjusted for all above-mentioned factors.

All covariates were significant when analyzed in models 2–4, and most but not all were significant in the final adjusted model. All covariates but one (region) were separately significant in the base model.

Risk consumption of alcohol, estimated from the baseline questionnaire, was tested with the base model but HR was not increased and it did not change the estimate of MetS and therefore it was omitted. The item “ever shift work” (from the baseline questionnaire) tested with the base model was significantly increased but when tested with the set of work-related factors gave a P-value of 0.44 and was therefore omitted. The seven models were also performed for men and women separately. The same calculations were done for the subgroup (N=6352).

The SAS program version 9.4 (SAS Institute, Cary, NC, USA) was used for the analyses. For Cox regressions, the PHREG procedure in SAS was applied, and this was also used when proportionality of hazards was tested using log-log survival functions.

The Review board of the Regional Ethics Committee at the University of Gothenburg approved the study (082-15, 26 March 2015).

## Results

The entire study population consisted of 10 803 employed subjects (69.5% men, 30.5% women). Of these, 17.9% (21.5% of men, 9.7% of women) met the IDF criteria for MetS. Baseline characteristics and covariates of the study subjects are presented in table 1. Of the study population 41.9% had central obesity at the start of study, and of these 25.5% were obese (BMI>30). Of those with MetS, 65.4% were not obese (BMI>30). In the non-MetS group, 27.8% of men and 32.1% of women had a waist circumference exceeding the IDF criteria but did not fulfill the other criteria required for definition of MetS. Elevated FPG levels or treatment for diabetes were evident in 5.8% of subjects in the non-MetS group, but only 0.8% of them had a diabetes diagnosis.

**Table 1:** Baseline characteristics and covariates, by gender, of subjects in the non-metabolic syndrome (non-MetS) and metabolic syndrome (MetS) groups. [IDF=International Diabetes Federation; HDL-C=high-density lipoprotein cholesterol; BMI=body mass index; T=treatment].

	Men		Women		All		All (N=10 803)	N
		
	Non-MetS (N=5893)	MetS (N=1614)	Non-MetS (N=2977)	MetS (N=319)	Non-MetS (N=8870)	MetS (N=1933)		

	%	%	%	%	%	%	%	
IDF criteria for metabolic syndrome								
Central obesity, men >94 cm, women >80 cm	27.8	100	32.1	100	29.3	100	41.9	10 803
Triglycerides >1.7 mmol/L or T	18.4	82.3	3.4	60.5	13.3	78.7	25.0	10 803
HDL-C, men, <1.0 mmol/L, women, <1.3 mmol/L or T	8.7	42.6	10.8	66.1	9.4	46.5	16.0	10 803
Hypertension, systolic >130 mmHg, diastolic >85 mmHg or T	31.3	78.3	22.2	77.4	28.3	78.1	37.2	10 803
Fasting glucose, >5.6 mmol/L or diagnosed diabetes	7.3	39.9	2.8	33.9	5.8	38.9	11.7	10 803
Covariates								
Diabetes diagnosis	0.9	4.8	0.5	6.6	0.8	5.1	1.6	10 747
Obesity (BMI >30)	5.3	34.2	6.3	37.0	5.6	34.6	10.8	10 800
Counties of Västernorrland and Jämtland, Sweden	53.7	69.3	24.1	49.2	43.7	66.0	47.7	10 803
Job level: low and medium	62.2	66.0	60.1	74.8	61.5	67.5	62.6	10 713
Education level: low (<10 years)	16.8	26.5	12.7	21.6	15.4	25.7	17.3	10 803
Education level: high (university level or similar)	24.6	14.7	37.3	21.9	28.9	15.9	26.5	10 803
Civil status: single	25.2	23.4	28.4	21.6	26.2	23.1	25.7	10 777
Smoking habits: ever-smoking	47.4	62.2	53.2	59.6	49.4	61.8	51.6	10 535
Leisure-time physical activity: rarely or never	23.8	37.8	19.9	30.8	22.5	36.7	25.0	10 769
Leisure-time physical activity: regularly	36.6	20.9	46.6	29.9	39.9	22.3	36.8	10 769
Low job decision latitude according to the Karasek model	54.3	59.3	56.9	65.5	55.2	60.3	56.1	9974
Physical workload at work: low	35.6	32.4	50.4	43.6	40.5	34.2	39.1	10 738
Physical workload at work: high	14.1	11.0	9.7	12.5	12.6	11.3	12.4	10 738

The results from baseline measurements and blood samples for study subjects with or without MetS are presented in table 2. Age at inclusion was about 6 years lower in the non-MetS group (P<0.05). Measurements and blood samples displayed elevated levels of triglycerides, FPG and systolic and diastolic BP, and lower levels of HDL-C in the MetS group compared with the non-MetS group, as expected.

**Table 2:** Baseline measurements and age, by gender, of subjects in the non-metabolic syndrome (non-MetS) and metabolic syndrome (MetS) groups. [SD=standard deviation; HDL-C=high-density lipoprotein cholesterol]

	Men				Women				All				Total	
			
	Non-MetS (N=5893)		MetS (N=1614)		Non-MetS (N=2977)		MetS (N=319)		Non-MetS (N=8870)		MetS (N=1933)		All workers (N=10 803)	
						
	Mean	SD	Mean	SD	Mean	SD	Mean	SD	Mean	SD	Mean	SD	Mean	SD
Age at inclusion in the study, years	41.4	10.9	46.9 ^a^	9.4	41.4	10.7	48.6 ^a^	8.9	41.4	10.8	47.1 ^a^	9.3	42.4	10.8
Central obesity, waist circumference, cm	89.7	8.8	103.3	7.9	76.9	9.2	93.7	10.2	85.4	10.8	101.7	9.1	88.3	12.2
Triglycerides, mmol/L	1.3	0.8	2.5	1.5	0.9	0.4	1.9	1.0	1.1	0.7	2.4	1.4	1.4	1.0
HDL-C, mmol/L	1.4	0.3	1.1	0.3	1.7	0.4	1.3	0.3	1.5	0.4	1.2	0.3	1.4	0.4
Blood pressure, systolic, mmHg	123	13.9	136	16.3	118	14.4	136	17.7	121	14.3	136	16.6	124	15.7
Blood pressure, diastolic, mmHg	74	9.8	82	10.6	72	9.5	80	10.0	73	9.8	82	10.5	75	10.4
Fasting plasma glucose (FPG), mmol/L	5.2	0.8	6.0	1.6	4.9	0.7	5.9	1.8	5.1	0.8	5.9	1.6	5.3	1.1

a P<0.05

Table 3 illustrates the proportion of subjects receiving all-cause disability pension as well as the mean age at disability pension and the incidence rate of all-cause disability pension. Total person-years at risk were 149 938, with a mean of 14.4 (SD 5.3) years for the non-MetS group and 11.4 (SD 5.5) years for the MetS group (data not shown). All-cause disability pension occurred in 7.5% of men in the non-MetS group compared with 15.2% in the MetS group; for women, the corresponding figures were 12.7% and 23.2%, respectively. The incidence rate of all-cause disability pension for subjects in the non-MetS group was 6.4 per 1000 person-years and 14.5 for subjects with MetS. Mean age at all-cause disability pension was similar between men and women in the MetS group but not in the non-MetS group. The mean age at all-cause disability pension for all subjects in the study was 55.4 years of age (SD 7.5).

**Table 3:** Incidence of all-cause disability pension during follow-up among subjects in the non-metabolic syndrome (non-MetS) and metabolic syndrome (MetS) groups, incidence rate per 1000 person-years (IR/1000 py), with 95% confidence intervals (CI). [SD=standard deviation].

	Subjects	Time at risk	All-cause disability pension		Age at disability pension, years		Incidence rate	
				
	N	Person-years	N	%	Mean	SD	IR/1000 py	95% CI
All subjects	10 803	149 938	1137	10.5	55.4	7.5	7.6	7.1–8.0
Non-MetS	8870	127 836	817	9.2	55.0	7.8	6.4	6.0–6.8
MetS	1933	22 102	320	16.6	56.4	6.3	14.5	12.9–16.2
Non-MetS men	5893	83 940	440	7.5	55.9	7.3	5.2	4.8–5.8
MetS men	1614	18 733	246	15.2	56.5	6.1	13.1	11.5–14.8
Non-MetS women	2977	43 896	377	12.7	53.9	8.3	8.6	7.7–9.5
MetS women	319	3369	74	23.2	56.0	6.9	22.0	17.2–27.6

Table 4 displays the risk of all-cause disability pension among subjects with MetS, with adjustment for potential explanatory and mediating factors at baseline. After adjustment for all covariates the final HR for all-cause disability pension in the MetS group subjects was 1.37 (95% CI 1.18–1.60) compared with the non-MetS group. Socio-demographic factors, health behaviors and work-related factors all influenced the risk for all-cause disability pension. However, the increased risk for all-cause disability pension remained elevated in the MetS group when adjusting for these factors. The final HR for women will be 1.34 (95% CI 1.01–1.79) if we exclude the non-significant factors (P>0.20) in model 7 (data not shown).

**Table 4:** Risk of all-cause disability pension among subjects with metabolic syndrome (MetS), adjusted for potential explanatory and mediating factors at baseline. Risks are given as hazard ratios (HR) with 95% confidence intervals (CI).

Model	Men (N=7507)		Women (N=3296)		Total (N=10 803)	
		
	HR	95% CI	HR	95% CI	HR	95% CI
Age (and sex)-adjusted base model ^a^	1.66	1.41–1.94	1.58	1.23–2.04	1.63	1.43–1.87
Adjusted for socio-demographic factors ^b^	1.58	1.35–1.85	1.48	1.14–1.91	1.55	1.35–1.77
Adjusted for health behaviors ^c^	1.55	1.32–1.83	1.53	1.18–1.98	1.55	1.36–1.78
Adjusted for work-related factors ^d^	1.61	1.37–1.90	1.56	1.20–2.04	1.60	1.40–1.84
Adjusted for non-MetS-related factors ^e^	1.55	1.31–1.83	1.51	1.15–1.97	1.54	1.34–1.78
Adjusted for diabetes and obesity ^f^	1.44	1.21–1.71	1.35	1.02–1.78	1.41	1.22–1.63
Adjusted for all above factors ^g^	1.40	1.17–1.69	1.31	0.98–1.76	1.37	1.18–1.60

a Model 1: age (sex) at inclusion in the study.

b Model 2: age (sex), region, job level, low education, university education, civil status (all factors significant).

c Model 3: age (sex), ever-smoking, low exercise, regular exercise (all factors significant).

d Model 4: age (sex), low control (according to the demand-control model), low physical workload, high physical workload at work (all factors significant).

e Model 5: age (sex), region, job level, low education, university studies, civil status, ever-smoking, low exercise, regular exercise, low control at work (according to the demand-control model), low physical workload at work, high physical workload at work (most factors significant).

f Model 6: age (sex), diabetes and obesity (all factors significant).

g Model 7: age (sex), region, low education, university studies, civil status, ever-smoking, low exercise, regular exercise, low control at work (according to the demand-control model), low physical workload at work, high physical workload at work, diabetes and obesity (most factors significant).

With adjustment for diabetes and obesity in the base model, the HR for all-cause disability pension in the MetS group was 1.41 (95% CI 1.22–1.63). The risk for all-cause disability pension when having diabetes was 2.10 (95% CI 1.56–2.82) in this model (data not shown). The results were similar for men and women in all combinations of chosen covariates in the specified models.

The subgroup answering the follow-up questionnaire in 2009 had similar baseline characteristics and covariates as the whole study population (data not shown). In the base model for the subgroup the risk for all-cause disability pension was 1.84 (95% CI 1.53–2.21) in the MetS group. After adjusting for reported chronic diseases, HR was 1.52 (95% CI 1.23–1.89) and after adjusting for chronic diseases and all other covariates, HR was 1.32 (95% CI 1.04–1.68). For women in the MetS group, the risk for all-cause disability pension was non-significant when adjusting for reported chronic diseases in the base model, HR 1.21 (95% CI 0.81–1.81). Among men the corresponding analysis yielded an HR of 1.69 (95% CI 1.30–2.19).

## Discussion

The main finding in this working life prospective study is that Swedish employees with MetS had a considerably elevated risk of all-cause disability pension compared with employees without MetS. The risk for all-cause disability pension remained elevated after adjustment for other covariates such as age at inclusion in the study, sex, socio-demographic factors, health behaviors, work-related factors including the demand-control model, and the MetS-related factors diabetes and obesity. To our knowledge, this is the first study on this topic.

The presence of MetS or non-MetS was based on an internationally recognized definition of MetS, the IDF criteria, which is clinically useful (21). Measurements of waist circumference, BMI, BP, blood samples of FPG and blood lipids are easily accessible in everyday medical practice. The IDF criteria also allow comparison with the results from other studies using the same or comparable MetS criteria. This study found similar results, concerning MetS, as reported in previous studies in which the prevalence was estimated to be about 10–20%, depending on the criteria definition for MetS, as well as the population and the subjects’ age (3).

Our study is based on a long-term follow-up of a representative cohort of employed men and women of normal working age, recruited from a variety of occupations at workplaces located in three counties in Sweden. Data collection in the WOLF surveys was based on self-administered questionnaires and closely supervised by local occupational health service personnel who also performed standardized measurements and blood sampling. The assessment of MetS was based on at least one health examination carried out in the WOLF cohort at each subject’s entrance year into the study. The health examination included the necessary measurements and blood samples to confirm or reject the MetS diagnosis. The assessment of self-reported chronic disease or conditions of importance was first established in the final survey questionnaire in 2009. The quality of data in the LISA database depends on the registries that supply data to the database; and the linkage between these registers is by use of the individual PIN code as a common denominator (20).

Since the 2009 WOLF survey did not comprise measurements and blood sampling, the true prevalence of MetS in the cohort at the end of the study period is not known even though this study shows that one-third of subjects in the non-MetS group met the prerequisite for MetS, increased waist circumference, but did not fulfill the other MetS criteria at baseline.

The main focus in this study was working employees; those on long-term leave from their workplace were excluded. The size of the total workforce is therefore not known, which may have led to a possible underestimation of the risk of all-cause disability pension.

The complex mechanisms in MetS leading to reduced work ability and, eventually, the loss of work ability are not fully understood. Insulin resistance, visceral adiposity and atherogenic dyslipidemia are known interrelated features sharing common mediators and pathophysiological mechanisms (2). We therefore wanted to adjust for diabetes and obesity in our analyses, but that did not account for the risk for disability pension among subjects with MetS.

Another aspect of work ability is the cognitive requirements in modern working life. In a complex working life, cognitive demands are crucial for productivity and could therefore be affected by the complex neuroanatomical and neuroendocrine changes inherent in MetS, as described by Taylor & MacQueen (13).

The chain of action explaining why MetS leads to an elevated risk of disability pension is to some extent understood concerning risk factors and mechanisms, but the complex underlying causes of loss of work ability are not known.

### Implications

In this study, we found an overall elevated risk of all-cause disability pension among study subjects with MetS. The implications of this new knowledge are that it could, through screening of groups with at least one risk factor, contribute to an awareness of the importance of early medical recognition of MetS, enabling necessary lifestyle changes, medical treatment and adequate intervention at the workplace in order to maintain work ability throughout the working life.

Contrary to MetS, DM2 is a well-known identifiable condition, which is often recognized early and monitored and medically treated, and whose impact on work ability and risk for disability pension is described in numerous studies (8–10). In this study, the number of subjects with MetS by far exceeded the number of subjects with diagnosed DM2; however, compared with DM2, MetS is much less of a diagnosed and medically treated entity. This is also understood by the fact that MetS is not recognized as a definable unique International Classification of Diseases, 10^th^ revision (ICD-10), diagnostic code; although the definition “metabolic syndrome” was established by WHO already in 1998 (1, 24). Today the risk factors related to metabolic disorders – obesity, abdominal obesity, blood lipid disturbances, hypertension, and insulin resistance with diabetic and non-diabetic hyper glycaemia – are often separately diagnosed and medically treated. A patient with hypertension or lipid disturbances is not routinely checked for waist circumference, and vice versa, but measurement of waist circumference is mandatory in establishing the entity of MetS, using the IDF criteria. These shortcomings complicate systematic preventive actions. The lack of a recognized diagnostic entity with an ICD-10 diagnostic code also implies that subjects with unidentified MetS are at risk of being without proper lifestyle interventions and medical care for the combined risk burden of the syndrome and also of work disability, and may eventually end their working life at an earlier stage, with a disability pension, as illustrated in this study.

### Concluding remarks

Many factors are involved in the risk of all-cause disability pension but the presence of MetS was an independent and significant risk factor in this study.
